# The Impact of Dietary Components on Regulatory T Cells and Disease

**DOI:** 10.3389/fimmu.2020.00253

**Published:** 2020-02-21

**Authors:** Rebeca Arroyo Hornero, Ibrahim Hamad, Beatriz Côrte-Real, Markus Kleinewietfeld

**Affiliations:** VIB Laboratory of Translational Immunomodulation, VIB Center for Inflammation Research (IRC), University of Hasselt, Hasselt, Belgium

**Keywords:** diet, microbiome, Treg—regulatory T cell, autoimmunity, environmental factors

## Abstract

The rise in the prevalence of autoimmune diseases in developed societies has been associated with a change in lifestyle patterns. Among other factors, increased consumption of certain dietary components, such as table salt and fatty acids and excessive caloric intake has been associated with defective immunological tolerance. Dietary nutrients have shown to modulate the immune response by a direct effect on the function of immune cells or, indirectly, by acting on the microbiome of the gastrointestinal tract. FOXP3^+^ regulatory T cells (Tregs) suppress immune responses and are critical for maintaining peripheral tolerance and immune homeostasis, modulating chronic tissue inflammation and autoimmune disease. It is now well-recognized that Tregs show certain degree of plasticity and can gain effector functions to adapt their regulatory function to different physiological situations during an immune response. However, plasticity of Tregs might also result in conversion into effector T cells that may contribute to autoimmune pathogenesis. Yet, which environmental cues regulate Treg plasticity and function is currently poorly understood, but it is of significant importance for therapeutic purposes. Here we review the current understanding on the effect of certain dietary nutrients that characterize Western diets in Treg metabolism, stability, and function. Moreover, we will discuss the role of Tregs linking diet and autoimmunity and the potential of dietary-based interventions to modulate Treg function in disease.

## Introduction

An appropriate balance between pro- and anti-inflammatory immune responses is required to protect organisms from invading pathogens and tumor development without incurring in autoimmune and allergic diseases. While different cell populations with anti-inflammatory activity have been identified, CD4^+^FOXP3^+^ regulatory T cells (Tregs) are the most well-defined. FOXP3 transcription factor determines Treg cell lineage and is essential for appropriate immune homeostasis. Loss-of-function mutations in *foxp3* lead to fatal immune disorders in humans (IPEX) (1, 2) and mice (Scurfy phenotype) (3).

Tregs suppress innate and adaptive immune responses using a broad array of molecular mechanisms which e.g., involve cell-contact dependent mechanisms (4), the release of soluble factors (5, 6), deprivation of growth factors (7), induction of apoptosis of target cells (8), and ATP hydrolysis and adenosine production (9, 10). Although there is versatility in the Treg response that allows for a specialized response according to the environment, the anatomical location, and the type of the cell to suppress (11, 12), increasing evidence suggests lack of Treg stability as a culprit of autoimmunity (13). Tregs isolated for instance from T1D (14, 15), MS (16–19) and SLE (20) patients showed acquisition of pro-inflammatory functions and reduced suppressive potency *in vitro*.

**Figure 1:**
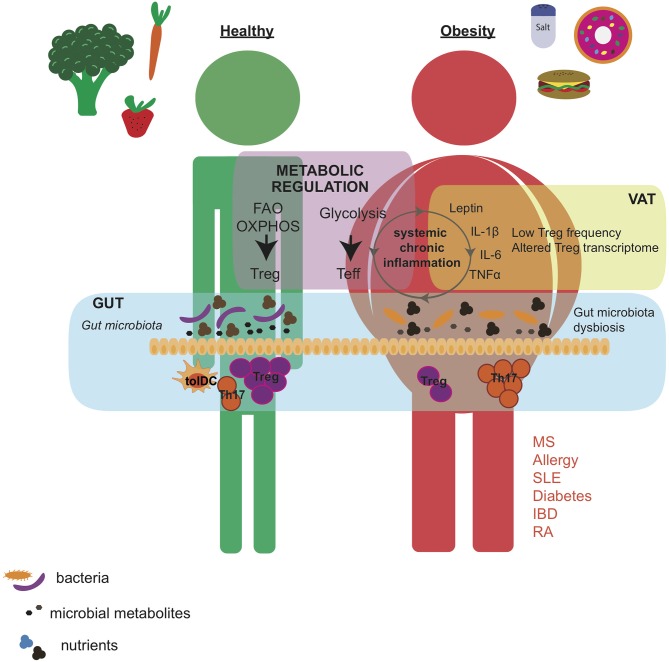
Western diet affects gut microbial composition and induces low-degree chronic inflammation that alters the metabolic status of the individual. Healthy diet supports the growth of bacterial species that, by the production of immunomodulatory metabolites, promotes Treg induction over Th17 cell development in the intestine. By contrast, western diet, characterized by high caloric intake, and high levels of salt and cholesterol, leads to obesity, increased secretion of pro-inflammatory adipokines and cytokines, and altered gut microbial composition. These changes are associated with an altered Treg phenotype and higher Th17 cell differentiation in the VAT and intestine. Importantly, Treg and Teff cells developed in the gut and VAT have a systemic effect and may contribute to exacerbation of autoimmune pathogenicity. Moreover, the frequency and function of Tregs may also be regulated by metabolic pathways such as FAO, OXPHOS, and glycolysis that depend on the nutritional state of the individual. VAT, visceral adipose tissue; Teff, effector T cell; FAO, fatty acid oxidation; OXPHOS, oxidative phosphorylation; MS, multiple sclerosis; SLE, systemic lupus erythematosus; IBD, inflammatory bowel disease; RA, rheumatoid arthritis.

Whereas, genetic factors clearly predispose to autoimmune development, the dramatic increase in the incidence of autoimmune diseases in Western countries suggests Western lifestyle patterns as important triggers of disease [reviewed in (21, 22)]. A variety of factors have been proposed to favor autoimmune development such as decrease pathogen exposure, smoke, hormones, stress, pollutants, dietary components and obesity (23–27). Moreover, increasing data highlight the complex interplay between nutrition, metabolic state and the immune response. Caloric restriction ameliorates disease severity and increases the lifespan in experimental animal models of inflammation and autoimmunity (28–30). By contrast, obesity is one of the most consisting factors that predispose for autoimmunity, having being linked with MS (31), T1D (32), psoriasis (33), and Chron's disease (34) (Figure 1). In addition, diet alters the gut microbial composition. Gut bacteria and their metabolites regulate pro-inflammatory and regulatory T cell responses in the gut, which could exert systemic effects in the individual (35–37).

Although there are many other cell types and environmental factors involved in triggering autoimmunity, given their crucial role in disease regulation, we will summarize the evidence provided by experimental and epidemiological studies associating nutrition, regulatory T cell function and autoimmunity.

## Treg Regulation and Heterogeneity

We and others have shown before that different cell subsets can be distinguished within the pool of Tregs (9, 38). Recent immune phenotyping by mass cytometry and single cell transcriptomic analysis have further demonstrated the heterogeneity of the FOXP3^+^ Treg population (39, 40). Therefore, these technologies could potentially aid in the identification of novel markers involved in Treg function, stability and migration and in gaining a better understanding of Treg biology. Tregs are typically categorized according to their origin into two subsets; those that develop in the thymus (tTregs) as a distinct cell lineage, and those induced from CD4^+^CD25^−^FOXP3^−^ naive T cells in peripheral tissues (pTregs). *In vitro*, FOXP3^+^ Tregs can also be generated from CD4^+^FOXP3^−^ T cells by e.g., culturing them in the presence of TGF-β, IL-2, and anti-CD3 stimulation (41, 42) being generally named as iTregs, although their functional activity is not well-defined in humans.

FOXP3 is regulated at transcriptional and post-transcriptional level in response to environmental cues [reviewed in (43)]. Demethylation at specific regions of the *foxp3* locus is pivotal for regulating FOXP3 expression in different Treg subsets (44). Moreover, distinct FOXP3 splicing variants have been described in humans (45–49) and variations in their relative expression are present in autoimmune disease patients (50–54), suggesting a link between FOXP3 post-transcriptional regulation and autoimmune pathogenicity.

Signals driven by the cytokine milieu (55–59), co-stimulatory molecules (60–62) and the strength of the TCR signaling (63–65) allow Tregs to adapt to the immune environment through e.g., changes in FOXP3 expression. Several studies have shown that, under certain inflammatory conditions, some Tregs secrete pro-inflammatory cytokines and lose their suppressive function (13, 66–72). Interestingly, phenotypically distinct Treg subsets in humans and mice have been described that mirror CD4^+^ Th cell populations by specific co-expression of chemokine receptors, cytokines, and lineage specifying-transcription factors classically associated with Th cells (18, 73–76). The acquisition of Th-specific markers may allow Tregs to co-localize and regulate particular Th cell subsets *in vivo* (76). However, it might also be an indication of loss of function. Indeed, an increase in IFN-γ-producing Tregs has been associated with e.g., T1D, MS and autoimmune hepatitis (15, 18, 77). Also, the frequency of Tregs expressing IL-17 is increased in e.g., human patients with psoriasis, IBD and RA (62, 78–83). These data suggest that some Th-like Tregs may lose their ability to suppress immune responses and, instead, may contribute to autoimmunity.

Additionally, Tregs show phenotypical differences depending on which tissue they reside in, with the best non-lymphoid-tissue Treg populations described being those residing in visceral adipose tissue (VAT), skeletal muscle, colonic lamina propria and skin [reviewed in (84–86)]. In general, tissue-resident Tregs are characterized by higher frequency, self-antigen TCR repertoire with clonal micro-expansion, and a specific transcriptional profile different from Tregs in lymphoid organs (87–90). Moreover, by the use of single cell transcriptomics it was revealed that Tregs are highly homogenous within each tissue (40). These distinct phenotypes allow for cell accumulation in specific tissues and dedicated function within the microenvironment [reviewed in (84)].

The existence of multiple Treg subsets with specialized function dependent on environmental signals shows the complexity of Treg biology, but it also makes Treg plasticity and function susceptible to pharmaceutical intervention. Whether changes in diet or microbial composition associated with a Western lifestyle can control Treg function is being actively studied.

## Obesity and Vat Tregs

Excessive calories are stored as fat in adipose tissue, which also acts as an “endocrine organ” releasing pro-inflammatory adipokines and cytokines such as TNF-α, IL-6, IL-1β, and leptin, resulting in systemic low-degree chronic inflammation (91, 92) (Figure 1). Multiple immune cell types reside in the adipose tissue and play a role in inflammation and metabolic dysregulation (87, 93–99). In particular, obese mice display a dramatic reduction in Treg numbers specifically in adipose tissue, but not in other fat depots, nor in other non-lymphoid tissues and spleen or lymph nodes (87, 100). Cytokines and adipokines may be involved in controlling Treg fluctuations in obese VAT. Obesity positively correlates with IL-6 and IL-17 expression in mice and humans (101, 102). IL-6 promotes Th17 over Treg development (103) and obese-induced Th17 cell expansion was correlated with exacerbated disease symptoms in autoimmune disease models of experimental autoimmune encephalomyelitis (EAE) and colitis (102, 104). Leptin favors Th1 responses (105–109) and Th17 differentiation (110), but inhibits Treg proliferation (111). Moreover, leptin deficient mice showed a decrease in pathogenic inflammation in most of experimental models of IBD (112), RA (113, 114) and MS (108, 109). Similarly, relapsing-remitting MS patients also displayed an inverse correlation between frequency of Tregs and serum leptin levels (115), indicating that leptin may act as a link between obesity, Treg numbers and immunological tolerance.

In addition to differences in frequency, it was demonstrated that VAT-Tregs isolated from genetically promoted (leptin-deficient) or diet-induced (high fat chow) insulin-resistant mouse models of obesity have an altered transcriptional signature compared to lean mice (116). Phenotypical changes driven by obesity highlight the adaptability of VAT-Tregs to metabolic perturbations and suggest that obesity might alter Treg plasticity. Although most of the molecular mechanisms still need to be elucidated, it has been shown that excessive caloric intake leads to a dysregulation of intracellular nutrient-energy-sensing pathways and metabolic overload in immune cells (117, 118).

## Metabolic Regulation of Tregs

Cellular metabolism regulates cell development, proliferation and function and is controlled by environmental cues and nutrient availability (119, 120). Tregs have a specific metabolic profile, which is mainly dependent on mitochondrial metabolism through fatty acid oxidation (FAO) or pyruvate dependent oxidative phosphorylation (OXPHOS) (121–125). mTOR, one of the main pathways linking nutritional availability with cellular activity, promotes glycolysis (126, 127) and regulates differentiation of Th1, Th17, and Tregs (128–130). Studies showed that *in vitro* over-activation of mTOR, by culturing in media containing high concentration of nutrients or leptin, impaired Treg proliferation and the induction of FOXP3 expression (131). Treatment with rapamycin or neutralizing anti-leptin mAb reversed this effect and resulted in increased Treg frequencies and lessened EAE severity. However, continuous treatment with rapamycin or genetic mTOR silencing impeded Treg proliferation in the long term *in vivo* (131). Hence, periods of high and low nutrient levels, required for oscillatory changes in mTOR activity, may be necessary for Treg homeostasis and immunotolerance [reviewed in (132)].

Deletion of PTEN, a negative regulator of PI3K, also contributes to Treg regulation by enhancing glycolysis, decreasing FOXP3 expression and inducing the generation of effector T cells (133, 134). Additionally, the metabolic sensor LKB1 acts through AMPK promoting OXPHOS over glycolysis, and its deletion on Tregs led to alterations in cellular metabolism and the development of autoimmune diseases associated with dampened FOXP3 expression (124, 135). On the other hand, AMPK is considered an antagonist of mTOR activity with the ability to promote FAO (136, 137). Berod et al. showed that deletion of ACC1, a key enzyme in fatty acid synthesis, promoted AMPK activity in CD4^+^ T cells leading to increases in FAO and Treg development, and ACC1 inhibition under EAE conditions improved disease severity by increasing Treg/Th17 ratio (138).

Vitamins and indoles also modulate Treg function (59). For example, retinoic acid (vitamin A metabolite) acts in conjunction with TGF-β promoting the induction of Tregs from naive T cells and stabilizing FOXP3 expression, which prevented their conversion into Th1/Th17 cells in the presence of IL-1β/IL-6 (139, 140). Calcitriol, a vitamin D metabolite, enhanced the growth of Tregs (141, 142). Vitamin C has been found to increase the generation of FOXP3^+^ iTregs on alloantigen-specific Treg induction cultures and to cause a pronounced TSDR demethylation, resulting in an elevated FOXP3 stability (143). Interestingly, vitamin C treatment may act in a distinct manner on tTreg and iTreg function. A recent study by Oyarce et al. showed that tTreg cells pretreated with vitamin C before coculturing with effector CD4^+^ T cells did not enhanced Treg ability to suppress T cell proliferation regardless of their increased FOXP3 expression. By contrast, *in vitro*-induced iTregs generated in presence of vitamin C showed improved suppressive capacities (144).

Metabolites associated with aryl hydrogen receptor (AHR) also control Treg function. Kynurenine is important for the generation, expansion and function of Tregs (145–147) and indole-3-carbanole (I3C) and 3,3′-diindolylmethane (DIM) promoted Treg infiltration to the CNS under EAE conditions, improving disease severity, and progression (147).

These data highlight the potential of targeting Treg metabolism to ameliorate autoimmune disease progression. However, more research regarding the therapeutic level of such modulation still has to be conducted. Besides, the limitations associated with studying Treg metabolism, due to their plasticity, culturing method, and biological source prompt a big challenge in the field.

## The Effect of Salt in Treg Plasticity

Increased intake of salt that is also common in Western diets has been linked with cardiovascular disease (148, 149) and autoimmunity (22, 150–154). Although the specific mechanisms are still being revealed, several studies in murine EAE and colitis models have demonstrated that elevated NaCl intake could exacerbate disease by promoting the induction of pathogenic Th17 cells via the SGK1-FOXO1 pathway (35, 36, 155). Besides, Wu et al. have recently described a direct effect of SGK1-FOXO1 in controlling Treg function, such as SGK1 deficiency in Tregs protected mice from the development of autoimmunity (156).

High-salt also induced secretion of IFN-ɤ and repressed IL-10 expression in Tregs, which resulted in impaired suppressive function *in vitro* and *in vivo* (155, 157, 158). In mice fed with high-salt diet, Tregs secreted more IFN-γ and failed to control colitis and xenogeneic GvHD, which was dependent on SGK1 signaling (157). IFN-γ-secreting FOXP3^+^ Tregs with reduced IL-10 expression have been found in MS and T1D patients and are thought to contribute to disease (15, 18, 158). Interestingly, the imbalance between IFN-γ- and IL-10-expressing-Tregs in MS patients was also observed when Tregs from healthy donors were exposed to high-salt *in vitro* (158), suggesting that a high-salt environment could skew Tregs toward a dysfunctional state. Moreover, PTGER2 and β-catenin appeared as upstream regulators of the SGK1-FOXO1 axis in response to high-salt concentration, and constitutive expression of active β-catenin in Tregs caused the development of Scurfy-like autoimmunity (158). Interestingly, stabilized active β-catenin has been also found in MS patients (158), suggesting that similar regulation may exist in humans. Recently, Luo et al. have proposed that, while high-salt alters the function of tTregs, it has no effect on pTregs or iTreg, which maintained unaltered transcriptional signature and stable FOXP3 expression, cytokine profile and suppressive function under high salt conditions (159). However, Wu et al. reported higher FOXP3 expression and regulatory function in iTregs and pTregs that lack SGK1 function (156), indicating that iTregs and pTregs could also be affected by high-salt via SGK1-FOXO1 axis. More studies are therefore required to clarify the role of high salt in Treg subpopulations.

Based on these findings, multiple studies have attempted to study the relation between salt intake and autoimmune disease in humans. In MS patients, Farez et al. have reported a positive correlation between disease activity and increased dietary sodium intake (150). Moreover, higher sodium concentration was observed in acute MS lesions than in chronic lesions (160). However, studies in larger cohorts have shown no significant correlation between salt consumption and the risk of MS development (161) or disease severity (162). Limitations in accurate measurement of NaCl levels and in the identification of the specific effect of salt independently of other dietary components may account for these controversial results [as discussed in (163)]. Since moderate increases in salt intake has proven to affect human immune cells, including T cells *in vivo* (26, 37), more specific analysis are needed to establish the role of NaCl in human autoimmune disease.

## The Gut Microbiota Affects Disease-Controlling Tregs

The human gastrointestinal tract is the major reservoir of microorganisms including bacteria, microeukaryotes, archaea, and viruses, all of which collectively constitute the commensal microbiota (164). Extensive research has demonstrated the intimate crosstalk between commensal microbiota and immune balance. Tregs residing in the intestine are critical for maintaining intestinal immune homeostasis (165–167). Increasing evidence shows that Tregs in the colonic lamina propria are mostly peripherally-induced and depend on microbiota-derived signals for proper development and function (90, 168, 169). In fact, germ-free mice or antibiotic-treated mice show a substantial reduction in colonic Treg frequency (168, 170, 171). Interestingly, induction of pTregs in the small intestine appears more dependent on dietary antigens than on microbial signals (172). By investigating germ-free mice fed on an antigen-free diet, Kim et al. elegantly showed reduction in pTreg numbers in small intestinal lamina propria compared to germ-free mice fed on conventional diet. Importantly, pTregs residing in small intestinal lamina propria suppressed immune responses against dietary antigens (172).

Haghikia et al. elegantly demonstrated that dietary fatty acids profoundly impact T cell subset differentiation in the gut, which had a subsequent impact on central nervous system autoimmunity. The authors showed that SCFAs increased Treg proliferation while long-chain fatty acids (LCFAs) supported Th1 and Th17 differentiation in the gut, which had a significant effect in EAE severity (173). The short-chain fatty acid (SCFA) butyrate, derived from fermentation of dietary carbohydrates by gut microbiota, is an important promotor of colonic Treg differentiation through epigenetic modifications in the *foxp3* locus, which induces FOXP3 expression and pTreg conversion (171, 174–176). *Clostridia* are known to produce high levels of butyrate and colonization of germ-free mice with these bacteria increased colonic Treg frequency and protected mice from colitis (177). By contrast, segmented filamentous bacteria (SFB) induced Th17 cell development in the gut promoting systemic autoimmunity (178–180). In a recent study, Luu et al. have shown that the SCFA pentanoate inhibited SFB-promoted Th17 cell induction by metabolic and epigenetically reprogramming CD4^+^ T cells to suppress IL-17 production and fostering IL-10 production in CD4^+^ T cells and B cells (181). Furthermore, Häger et al. reported increased Treg numbers in 36 RA patients after receiving high-fiber dietary supplementation for 28 days, which correlated with a higher Th1/Th17 ratio and decreased expression of markers associated with bone erosion (182).

Several studies have shown that administration of probiotic bacteria containing members of the *Lactobacillus, Streptococcus*, and *Bifidobacterium* genera primed DCs to induce the development of FOXP3^+^ Tregs and IL-10-secreting regulatory T cells (183, 184) (Figure 1). Poutahidis et al. showed that mice fed with Westernized “fast food”-style chow developed obesity and had increased IL-17 levels. By contrast, the addition of probiotic yogurt containing *Lactobacillus reuteri* into the diet was sufficient to induce weight loss by a Treg dependent mechanism (174). Importantly, diet alters the gut microbiome (185–187) and dysregulation of intestinal microbiota is associated with autoimmunity [reviewed in (188, 189)]. Wilck et al. have shown that increased salt consumption affects intestinal bacterial composition in mice and humans. *Lactobacillus* spp. was suppressed in high salt condition, but its supplementation prevented high salt-induced Th17 differentiation and ameliorated salt-sensitive hypertension and EAE severity (37, 154). Cekanaviciute et al. found that MS patients have a high presence of the *Akkermansia calcoaceticus* and *Akkermansia muciniphila*, and the exposure of healthy donor PBMC to these bacteria impaired Treg conversion while enhancing Th1 differentiation (190). These data connect diet with microbiota composition and autoimmune pathogenesis, raising the potential of microbiota-targeted therapies.

## Diet as a Therapeutic Aid to Control Autoimmunity

It is becoming clear that nutrition, metabolic state, microbiota, and autoimmunity are deeply interconnected. In addition to genetic factors, the Western diet characterized by high caloric intake in the form of processed food enriched in protein, sugar, fat and salt, is widely believed to contribute to the rise in autoimmune diseases in the last decades (Figure 1). However, one of the major challenges in investigating the effect of diet in human health is the impossibility to address the role of individual nutrients, which maybe the reason why a definite association between dietary interventions and outcomes in human autoimmune disease has not been established yet. Besides, dietary nutrients and microbial metabolites alter the immune response by acting on different immune cell populations, challenging our aim to identify underlying immunological mechanisms targeted during dietary interventions. For instance, we have recently corroborated that high salt diet lead to alterations in T cell populations in murine tumor transplantation models (191). However, inhibition of tumor growth given by high salt diet was largely independent of T cells in these models. Instead, high salt blocked the suppressive function of myeloid derived suppressor cells (MDSCs) *in vitro* and seems to promote thereby more pronounced anti-tumor immunity *in vivo* (191).

Obesity alters the balance between pro-inflammatory and suppressive T cells responses in adipose tissue, with Tregs losing their phenotypic identity and function (116), and resulting in break of self-tolerance (131) (Figure 1). Caloric restriction exerts immunoregulatory effects but is not suitable as general therapy for humans. Interestingly, Cignarella et al. have recently reported that intermittent fasting also improves disease outcomes in the EAE model as caloric restriction does (192). This effect was partially mediated by changes in the gut microbiota, since microbiota transplantation from mice under intermittent fasting into normally-fed mice could induce protection from EAE (192). Microbiota is a major determinant in the regulation of pro-inflammatory and regulatory T cell plasticity in the gut (35–37). Importantly, gut-resident T cells have the ability to traffic between different organs and exert a systemic effect in the organism (193, 194). Furthermore, these findings were translated into a small trial studying 16 MS patients that were on intermittent fasting for 15 days. Although no significant changes in gut bacteria composition was observed, a trend toward increased abundance of the Treg-inducer *Clostridia* bacteria was reported (177, 192).

As indicated by these data, dietary interventions and the use of probiotics may aid in the control of Treg stability and function by altering the milieu in which Tregs act *in vivo*, and help to restore immune responses in individuals with autoimmune prone Western lifestyle.

## Concluding Remarks

Although it is clear that Treg function is frequently altered in human autoimmunity, it should be noted that Tregs are a heterogenous population with distinct tissue-specific features, multiple functions and differential degree of plasticity in response to environmental cues. Moreover, autoimmune diseases are highly heterogenous and it is likely that different defects in Treg-mediated regulation are involved in different types of autoimmune disease and even in each individual depending on the specific genetic background (195). Increasing progress in purifying and subdividing Treg subsets and defining the mechanisms that dictate their function and plasticity will likely contribute to a better understanding on the role of Tregs in autoimmunity.

Dietary factors, via direct effects on immune cells or by acting indirectly through modulation of the gut microbiota, may regulate Treg plasticity and function and, therefore, may have the potential to control disease outcome. However, more research and tightly controlled studies are needed to assess the impact of specific dietary nutrients and bacteria or microbial metabolites on Tregs, autoimmunity, and human health.

## Author Contributions

RA, IH, BC-R, and MK wrote the manuscript.

### Conflict of Interest

The authors declare that the research was conducted in the absence of any commercial or financial relationships that could be construed as a potential conflict of interest.

## References

[1] Bennett CL Christie J Ramsdell F Brunkow ME Ferguson PJ Whitesell L. The immune dysregulation, polyendocrinopathy, enteropathy, X-linked syndrome (IPEX) is caused by mutations of FOXP3. Nat Genet. (2001) 27:20–1. 10.1038/83713 11137993

[2] Wildin RS Ramsdell F Peake J Faravelli F Casanova JL Buist N. X-linked neonatal diabetes mellitus, enteropathy and endocrinopathy syndrome is the human equivalent of mouse scurfy. Nat Genet. (2001) 27:18–20. 10.1038/83707 11137992

[3] Brunkow ME Jeffery EW Hjerrild KA Paeper B Clark LB Yasayko SA. Disruption of a new forkhead/winged-helix protein, scurfin, results in the fatal lymphoproliferative disorder of the scurfy mouse. Nat Genet. (2001) 27:68–73. 10.1038/83784 11138001

[4] Qureshi OS Zheng Y Nakamura K Attridge K Manzotti C Schmidt EM. Trans-endocytosis of CD80 and CD86: a molecular basis for the cell-extrinsic function of CTLA-4. Science. (2011) 332:600–3. 10.1126/science.1202947 21474713 PMC3198051

[5] Asseman C Mauze S Leach MW Coffman RL Powrie F. An essential role for interleukin 10 in the function of regulatory T cells that inhibit intestinal inflammation. J Exp Med. (1999) 190:995–1004. 10.1084/jem.190.7.995 10510089 PMC2195650

[6] Banchereau J Pascual V O'Garra A. From IL-2 to IL-37: the expanding spectrum of anti-inflammatory cytokines. Nat Immunol. (2012) 13:925–31. 10.1038/ni.2406 22990890 PMC3609707

[7] Barthlott T Moncrieffe H Veldhoen M Atkins CJ Christensen J O'Garra A. CD25+ CD4+ T cells compete with naive CD4+ T cells for IL-2 and exploit it for the induction of IL-10 production. Int Immunol. (2005) 17:279–88. 10.1093/intimm/dxh207 15684039

[8] Grossman WJ Verbsky JW Barchet W Colonna M Atkinson JP Ley TJ. Human T regulatory cells can use the perforin pathway to cause autologous target cell death. Immunity. (2004) 21:589–601. 10.1016/j.immuni.2004.09.002 15485635

[9] Borsellino G Kleinewietfeld M Di Mitri D Sternjak A Diamantini A Giometto R. Expression of ectonucleotidase CD39 by Foxp3+ Treg cells: hydrolysis of extracellular ATP and immune suppression. Blood. (2007) 110:1225–32. 10.1182/blood-2006-12-064527 17449799

[10] Bodor J Bopp T Vaeth M Klein M Serfling E Hunig T. Cyclic AMP underpins suppression by regulatory T cells. Eur J Immunol. (2012) 42:1375–84. 10.1002/eji.201141578 22678893

[11] Sakaguchi S Wing K Onishi Y Prieto-Martin P Yamaguchi T. Regulatory T cells: how do they suppress immune responses? Int Immunol. (2009) 21:1105–11. 10.1093/intimm/dxp095 19737784

[12] Tang Q Bluestone JA. The Foxp3+ regulatory T cell: a jack of all trades, master of regulation. Nat Immunol. (2008) 9:239–44. 10.1038/ni1572 18285775 PMC3075612

[13] Zhou X Bailey-Bucktrout SL Jeker LT Penaranda C Martinez-Llordella M Ashby M. Instability of the transcription factor Foxp3 leads to the generation of pathogenic memory T cells *in vivo*. Nat Immunol. (2009) 10:1000–7. 10.1038/ni.1774 19633673 PMC2729804

[14] Lawson JM Tremble J Dayan C Beyan H Leslie RD Peakman M. Increased resistance to CD4+CD25hi regulatory T cell-mediated suppression in patients with type 1 diabetes. Clin Exp Immunol. (2008) 154:353–9. 10.1111/j.1365-2249.2008.03810.x 19037920 PMC2633239

[15] McClymont SA Putnam AL Lee MR Esensten JH Liu W Hulme MA. Plasticity of human regulatory T cells in healthy subjects and patients with type 1 diabetes. J Immunol. (2011) 186:3918–26. 10.4049/jimmunol.1003099 21368230 PMC3091943

[16] Haas J Hug A Viehover A Fritzsching B Falk CS Filser A. Reduced suppressive effect of CD4+CD25high regulatory T cells on the T cell immune response against myelin oligodendrocyte glycoprotein in patients with multiple sclerosis. Eur J Immunol. (2005) 35:3343–52. 10.1002/eji.200526065 16206232

[17] Viglietta V Baecher-Allan C Weiner HL Hafler DA. Loss of functional suppression by CD4+CD25+ regulatory T cells in patients with multiple sclerosis. J Exp Med. (2004) 199:971–9. 10.1084/jem.20031579 15067033 PMC2211881

[18] Dominguez-Villar M Baecher-Allan CM Hafler DA. Identification of T helper type 1-like, Foxp3+ regulatory T cells in human autoimmune disease. Nat Med. (2011) 17:673–5. 10.1038/nm.2389 21540856 PMC3675886

[19] Costantino CM Baecher-Allan CM Hafler DA. Human regulatory T cells and autoimmunity. Eur J Immunol. (2008) 38:921–4. 10.1002/eji.200738104 18395861 PMC2752283

[20] Vargas-Rojas MI Crispin JC Richaud-Patin Y Alcocer-Varela J Quantitative and qualitative normal regulatory T cells are not capable of inducing suppression in SLE patients due to T-cell resistance. Lupus. (2008) 17:289–94. 10.1177/0961203307088307 18413409

[21] Javierre BM Hernando H Ballestar E. Environmental triggers and epigenetic deregulation in autoimmune disease. Discov Med. (2011) 12:535–45. 22204770

[22] Manzel A Muller DN Hafler DA Erdman SE Linker RA Kleinewietfeld M. Role of “Western diet” in inflammatory autoimmune diseases. Curr Allergy Asthma Rep. (2014) 14:404. 10.1007/s11882-013-0404-6 24338487 PMC4034518

[23] Bach JF. The effect of infections on susceptibility to autoimmune and allergic diseases. N Engl J Med. (2002) 347:911–20. 10.1056/NEJMra020100 12239261

[24] Okada H Kuhn C Feillet H Bach JF The “hygiene hypothesis” for autoimmune and allergic diseases: an update. Clin Exp Immunol. (2010) 160:1–9. 10.1111/j.1365-2249.2010.04139.x PMC2841828 20415844

[25] Matveeva O Bogie JFJ Hendriks JJA Linker RA Haghikia A Kleinewietfeld M. Western lifestyle and immunopathology of multiple sclerosis. Ann N Y Acad Sci. (2018) 1417:71–86. 10.1111/nyas.13583 29377214 PMC5947729

[26] Willebrand R Kleinewietfeld M. The role of salt for immune cell function and disease. Immunology. (2018) 154:346–53. 10.1111/imm.12915 29465812 PMC6002217

[27] Procaccini C Carbone F Galgani M La Rocca C De Rosa V Cassano S. Obesity and susceptibility to autoimmune diseases. Expert Rev Clin Immunol. (2011) 7:287–94. 10.1586/eci.11.18 21595595

[28] Matsuzaki J Kuwamura M Yamaji R Inui H Nakano Y. Inflammatory responses to lipopolysaccharide are suppressed in 40% energy-restricted mice. J Nutr. (2001) 131:2139–44. 10.1093/jn/131.8.2139 11481408

[29] Muthukumar A Sun D Zaman K Barnes JL Haile D Fernandes G. Age associated alterations in costimulatory and adhesion molecule expression in lupus-prone mice are attenuated by food restriction with n-6 and n-3 fatty acids. J Clin Immunol. (2004) 24:471–80. 10.1023/B:JOCI.0000040918.92219.d1 15359106

[30] Piccio L Stark JL Cross AH. Chronic calorie restriction attenuates experimental autoimmune encephalomyelitis. J Leukoc Biol. (2008) 84:940–8. 10.1189/jlb.0208133 18678605 PMC2638732

[31] Mokry LE Ross S Timpson NJ Sawcer S Davey Smith G Richards JB. Obesity and multiple sclerosis: a mendelian randomization study. PLoS Med. (2016) 13:e1002053. 10.1371/journal.pmed.1002053 27351487 PMC4924848

[32] Ferrara PM Stoner L Cornwall J. Diagnosis of childhood obesity using BMI: potential ethicolegal implications and downstream effects. Obes Rev. (2017) 18:380–1. 10.1111/obr.12509 28098950

[33] Sterry W Strober BE Menter A International Psoriasis Council. Obesity in psoriasis: the metabolic, clinical and therapeutic implications. Report of an interdisciplinary conference and review. Br J Dermatol. (2007) 157:649–55. 10.1111/j.1365-2133.2007.08068.x 17627791

[34] Hass DJ Brensinger CM Lewis JD Lichtenstein GR. The impact of increased body mass index on the clinical course of Crohn's disease. Clin Gastroenterol Hepatol. (2006) 4:482–8. 10.1016/j.cgh.2005.12.015 16616354

[35] Kleinewietfeld M Manzel A Titze J Kvakan H Yosef N Linker RA. Sodium chloride drives autoimmune disease by the induction of pathogenic TH17 cells. Nature. (2013) 496:518–22. 10.1038/nature11868 23467095 PMC3746493

[36] Wu C Yosef N Thalhamer T Zhu C Xiao S Kishi Y. Induction of pathogenic TH17 cells by inducible salt-sensing kinase SGK1. Nature. (2013) 496:513–7. 10.1038/nature11984 23467085 PMC3637879

[37] Wilck N Matus MG Kearney SM Olesen SW Forslund K Bartolomaeus H. Salt-responsive gut commensal modulates TH17 axis and disease. Nature. (2017) 551:585–9. 10.1038/nature24628 29143823 PMC6070150

[38] Miyara M Yoshioka Y Kitoh A Shima T Wing K Niwa A. Functional delineation and differentiation dynamics of human CD4+ T cells expressing the FoxP3 transcription factor. Immunity. (2009) 30:899–911. 10.1016/j.immuni.2009.03.019 19464196

[39] Mason GM Lowe K Melchiotti R Ellis R de Rinaldis E Peakman M. Phenotypic complexity of the human regulatory T cell compartment revealed by mass cytometry. J Immunol. (2015) 195:2030–7. 10.4049/jimmunol.1500703 26223658

[40] Miragaia RJ Gomes T Chomka A Jardine L Riedel A Hegazy AN. Single-cell transcriptomics of regulatory T cells reveals trajectories of tissue adaptation. Immunity. (2019) 50:493–504 e7. 10.1016/j.immuni.2019.01.001 30737144 PMC6382439

[41] Abbas AK Benoist C Bluestone JA Campbell DJ Ghosh S Hori S. Regulatory T cells: recommendations to simplify the nomenclature. Nat Immunol. (2013) 14:307–8. 10.1038/ni.2554 23507634

[42] Shevach EM Thornton AM. tTregs, pTregs, and iTregs: similarities and differences. Immunol Rev. (2014) 259:88–102. 10.1111/imr.12160 24712461 PMC3982187

[43] Lu L Barbi J Pan F. The regulation of immune tolerance by FOXP3. Nat Rev Immunol. (2017) 17:703–17. 10.1038/nri.2017.75 28757603 PMC5793224

[44] Zheng Y Josefowicz S Chaudhry A Peng XP Forbush K Rudensky AY. Role of conserved non-coding DNA elements in the Foxp3 gene in regulatory T-cell fate. Nature. (2010) 463:808–12. 10.1038/nature08750 20072126 PMC2884187

[45] Allan SE Passerini L Bacchetta R Crellin N Dai M Orban PC. The role of 2 FOXP3 isoforms in the generation of human CD4+ Tregs. J Clin Invest. (2005) 115:3276–84. 10.1172/JCI24685 16211090 PMC1242190

[46] Smith EL Finney HM Nesbitt AM Ramsdell F Robinson MK. Splice variants of human FOXP3 are functional inhibitors of human CD4+ T-cell activation. Immunology. (2006) 119:203–11. 10.1111/j.1365-2567.2006.02425.x 17005002 PMC1782350

[47] Du J Huang C Zhou B Ziegler SF. Isoform-specific inhibition of ROR alpha-mediated transcriptional activation by human FOXP3. J Immunol. (2008) 180:4785–92. 10.4049/jimmunol.180.7.4785 18354202

[48] Kaur G Goodall JC Jarvis LB Hill Gaston JS Characterisation of Foxp3 splice variants in human CD4+ and CD8+ T cells–identification of Foxp3Delta7 in human regulatory T cells. Mol Immunol. (2010) 48(1–3):321–32. 10.1016/j.molimm.2010.07.008 20688398

[49] Mailer RK Joly AL Liu S Elias S Tegner J Andersson J. IL-1β promotes Th17 differentiation by inducing alternative splicing of FOXP3. Sci Rep. (2015) 5:14674. 10.1038/srep14674 26441347 PMC4593960

[50] Ryder LR Woetmann A Madsen HO Odum N Ryder LP Bliddal H. Expression of full-length and splice forms of FoxP3 in rheumatoid arthritis. Scand J Rheumatol. (2010) 39:279–86. 10.3109/03009740903555374 20476861

[51] Sambucci M Gargano F De Rosa V De Bardi M Picozza M Placido R. FoxP3 isoforms and PD-1 expression by T regulatory cells in multiple sclerosis. Sci Rep. (2018) 8:3674. 10.1038/s41598-018-21861-5 29487369 PMC5829149

[52] Joly AL Seitz C Liu S Kuznetsov NV Gertow K Westerberg LS. Alternative splicing of FOXP3 controls regulatory T cell effector functions and is associated with human atherosclerotic plaque stability. Circ Res. (2018) 122:1385–94. 10.1161/CIRCRESAHA.117.312340 29618596

[53] Joly AL Andersson J. Alternative splicing, FOXP3 and cardiovascular disease. Aging. (2019) 11:1905–6. 10.18632/aging.101897 30981208 PMC6503886

[54] Mailer RKW. Alternative splicing of FOXP3-virtue and vice. Front Immunol. (2018) 9:530. 10.3389/fimmu.2018.00530 29593749 PMC5859138

[55] Cheng G Yu A Malek TR. T-cell tolerance and the multi-functional role of IL-2R signaling in T-regulatory cells. Immunol Rev. (2011) 241:63–76. 10.1111/j.1600-065X.2011.01004.x 21488890 PMC3101713

[56] Tran DQ Ramsey H Shevach EM Induction of FOXP3 expression in naive human CD4+FOXP3 T cells by T-cell receptor stimulation is transforming growth factor-beta dependent but does not confer a regulatory phenotype. Blood. (2007) 110:2983–90. 10.1182/blood-2007-06-094656 17644734 PMC2018674

[57] Walker MR Kasprowicz DJ Gersuk VH Benard A Van Landeghen M Buckner JH. Induction of FoxP3 and acquisition of T regulatory activity by stimulated human CD4+CD25- T cells. J Clin Invest. (2003) 112:1437–43. 10.1172/JCI19441 14597769 PMC228469

[58] Sakaguchi S Vignali DA Rudensky AY Niec RE Waldmann H. The plasticity and stability of regulatory T cells. Nat Rev Immunol. (2013) 13:461–7. 10.1038/nri3464 23681097

[59] Hoeppli RE Wu D Cook L Levings MK. The environment of regulatory T cell biology: cytokines, metabolites, and the microbiome. Front Immunol. (2015) 6:61. 10.3389/fimmu.2015.00061 25741338 PMC4332351

[60] Francisco LM Salinas VH Brown KE Vanguri VK Freeman GJ Kuchroo VK. PD-L1 regulates the development, maintenance, and function of induced regulatory T cells. J Exp Med. (2009) 206:3015–29. 10.1084/jem.20090847 20008522 PMC2806460

[61] Stathopoulou C Gangaplara A Mallett G Flomerfelt FA Liniany LP Knight D. PD-1 inhibitory receptor downregulates asparaginyl endopeptidase and maintains Foxp3 transcription factor stability in induced regulatory T cells. Immunity. (2018) 49:247–63 e7. 10.1016/j.immuni.2018.05.006 30054205 PMC6105434

[62] Remedios KA Zirak B Sandoval PM Lowe MM Boda D Henley E. The TNFRSF members CD27 and OX40 coordinately limit TH17 differentiation in regulatory T cells. Sci Immunol. (2018) 3:eaau2042. 10.1126/sciimmunol.aau2042 30578350

[63] Sauer S Bruno L Hertweck A Finlay D Leleu M Spivakov M. T cell receptor signaling controls Foxp3 expression via PI3K, Akt, and mTOR. Proc Natl Acad Sci USA. (2008) 105:7797–802. 10.1073/pnas.0800928105 18509048 PMC2409380

[64] Ohkura N Hamaguchi M Morikawa H Sugimura K Tanaka A Ito Y. T cell receptor stimulation-induced epigenetic changes and Foxp3 expression are independent and complementary events required for Treg cell development. Immunity. (2012) 37:785–99. 10.1016/j.immuni.2012.09.010 23123060

[65] Li MO Rudensky AY. T cell receptor signalling in the control of regulatory T cell differentiation and function. Nat Rev Immunol. (2016) 16:220–33. 10.1038/nri.2016.26 27026074 PMC4968889

[66] Oldenhove G Bouladoux N Wohlfert EA Hall JA Chou D Dos Santos L. Decrease of Foxp3+ Treg cell number and acquisition of effector cell phenotype during lethal infection. Immunity. (2009) 31:772–86. 10.1016/j.immuni.2009.10.001 19896394 PMC2814877

[67] Tsuji M Komatsu N Kawamoto S Suzuki K Kanagawa O Honjo T. Preferential generation of follicular B helper T cells from Foxp3+ T cells in gut Peyer's patches. Science. (2009) 323:1488–92. 10.1126/science.1169152 19286559

[68] Kim HJ Barnitz RA Kreslavsky T Brown FD Moffett H Lemieux ME. Stable inhibitory activity of regulatory T cells requires the transcription factor Helios. Science. (2015) 350:334–9. 10.1126/science.aad0616 26472910 PMC4627635

[69] Polesso F Sarker M Anderson A Parker DC Murray SE. Constitutive expression of NF-kappaB inducing kinase in regulatory T cells impairs suppressive function and promotes instability and pro-inflammatory cytokine production. Sci Rep. (2017) 7:14779. 10.1038/s41598-017-14965-x 29116141 PMC5677020

[70] Yang XO Nurieva R Martinez GJ Kang HS Chung Y Pappu BP. Molecular antagonism and plasticity of regulatory and inflammatory T cell programs. Immunity. (2008) 29:44–56. 10.1016/j.immuni.2008.05.007 18585065 PMC2630532

[71] Kleinewietfeld M Hafler DA. The plasticity of human Treg and Th17 cells and its role in autoimmunity. Semin Immunol. (2013) 25:305–12. 10.1016/j.smim.2013.10.009 24211039 PMC3905679

[72] Kleinewietfeld M Hafler DA. Regulatory T cells in autoimmune neuroinflammation. Immunol Rev. (2014) 259:231–44. 10.1111/imr.12169 24712469 PMC3990868

[73] Haringer B Lozza L Steckel B Geginat J. Identification and characterization of IL-10/IFN-gamma-producing effector-like T cells with regulatory function in human blood. J Exp Med. (2009) 206:1009–17. 10.1084/jem.20082238 19414553 PMC2715038

[74] Gandhi R Kumar D Burns EJ Nadeau M Dake B Laroni A. Activation of the aryl hydrocarbon receptor induces human type 1 regulatory T cell-like and Foxp3(+) regulatory T cells. Nat Immunol. (2010) 11:846–53. 10.1038/ni.1915 20676092 PMC2929008

[75] Beriou G Costantino CM Ashley CW Yang L Kuchroo VK Baecher-Allan C. IL-17-producing human peripheral regulatory T cells retain suppressive function. Blood. (2009) 113:4240–9. 10.1182/blood-2008-10-183251 19171879 PMC2676084

[76] Duhen T Duhen R Lanzavecchia A Sallusto F Campbell DJ. Functionally distinct subsets of human FOXP3+ Treg cells that phenotypically mirror effector Th cells. Blood. (2012) 119:4430–40. 10.1182/blood-2011-11-392324 22438251 PMC3362361

[77] Arterbery AS Osafo-Addo A Avitzur Y Ciarleglio M Deng Y Lobritto SJ. Production of proinflammatory cytokines by monocytes in liver-transplanted recipients with *de novo* autoimmune hepatitis is enhanced and induces TH1-like regulatory T cells. J Immunol. (2016) 196:4040–51. 10.4049/jimmunol.1502276 27183637 PMC4874532

[78] Voo KS Wang YH Santori FR Boggiano C Wang YH Arima K. Identification of IL-17-producing FOXP3+ regulatory T cells in humans. Proc Natl Acad Sci USA. (2009) 106:4793–8. 10.1073/pnas.0900408106 19273860 PMC2653560

[79] Hovhannisyan Z Treatman J Littman DR Mayer L. Characterization of interleukin-17-producing regulatory T cells in inflamed intestinal mucosa from patients with inflammatory bowel diseases. Gastroenterology. (2011) 140:957–65. 10.1053/j.gastro.2010.12.002 21147109 PMC3049831

[80] Pesenacker AM Bending D Ursu S Wu Q Nistala K Wedderburn LR. CD161 defines the subset of FoxP3+ T cells capable of producing proinflammatory cytokines. Blood. (2013) 121:2647–58. 10.1182/blood-2012-08-443473 23355538 PMC3617631

[81] Bovenschen HJ van de Kerkhof PC van Erp PE Woestenenk R Joosten I Koenen HJ. Foxp3+ regulatory T cells of psoriasis patients easily differentiate into IL-17A-producing cells and are found in lesional skin. J Invest Dermatol. (2011) 131:1853–60. 10.1038/jid.2011.139 21654831

[82] Sanchez Rodriguez R Pauli ML Neuhaus IM Yu SS Arron ST Harris HW. Memory regulatory T cells reside in human skin. J Clin Invest. (2014) 124:1027–36. 10.1172/JCI72932 24509084 PMC3934172

[83] Li L Boussiotis VA. The role of IL-17-producing Foxp3+ CD4+ T cells in inflammatory bowel disease and colon cancer. Clin Immunol. (2013) 148:246–53. 10.1016/j.clim.2013.05.003 23773923 PMC3808980

[84] Panduro M Benoist C Mathis D. Tissue Tregs. Annu Rev Immunol. (2016) 34:609–33. 10.1146/annurev-immunol-032712-095948 27168246 PMC4942112

[85] Burzyn D Benoist C Mathis D Regulatory T cells in non-lymphoid tissues. Nat Immunol. (2013) 14:1007–13. 10.1038/ni.2683 24048122 PMC4708287

[86] Cipolletta D. Adipose tissue-resident regulatory T cells: phenotypic specialization, functions and therapeutic potential. Immunology. (2014) 142:517–25. 10.1111/imm.12262 24484282 PMC4107662

[87] Feuerer M Herrero L Cipolletta D Naaz A Wong J Nayer A Lean, but not obese, fat is enriched for a unique population of regulatory T cells that affect metabolic parameters. Nat Med. (2009) 15:930–9. 10.1038/nm.2002 19633656 PMC3115752

[88] Hall JA Bouladoux N Sun CM Wohlfert EA Blank RB Zhu Q. Commensal DNA limits regulatory T cell conversion and is a natural adjuvant of intestinal immune responses. Immunity. (2008) 29:637–49. 10.1016/j.immuni.2008.08.009 18835196 PMC2712925

[89] Kolodin D van Panhuys N Li C Magnuson AM Cipolletta D Miller CM. Antigen- and cytokine-driven accumulation of regulatory T cells in visceral adipose tissue of lean mice. Cell Metab. (2015) 21:543–57. 10.1016/j.cmet.2015.03.005 25863247 PMC4747251

[90] Bilate AM Bousbaine D Mesin L Agudelo M Leube J Kratzert A. Tissue-specific emergence of regulatory and intraepithelial T cells from a clonal T cell precursor. Sci Immunol. (2016) 1:eaaf7471. 10.1126/sciimmunol.aaf7471 28783695 PMC6296461

[91] Fantuzzi G. Adipose tissue, adipokines, and inflammation. J Allergy Clin Immunol. (2005) 115:911–9. 10.1016/j.jaci.2005.02.023 15867843

[92] Hotamisligil GS Shargill NS Spiegelman BM. Adipose expression of tumor necrosis factor-alpha: direct role in obesity-linked insulin resistance. Science. (1993) 259:87–91. 10.1126/science.7678183 7678183

[93] Weisberg SP McCann D Desai M Rosenbaum M Leibel RL Ferrante AW Jr. Obesity is associated with macrophage accumulation in adipose tissue. J Clin Invest. (2003) 112:1796–808. 10.1172/JCI200319246 14679176 PMC296995

[94] Lumeng CN Bodzin JL Saltiel AR. Obesity induces a phenotypic switch in adipose tissue macrophage polarization. J Clin Invest. (2007) 117:175–84. 10.1172/JCI29881 17200717 PMC1716210

[95] Lumeng CN Deyoung SM Bodzin JL Saltiel AR. Increased inflammatory properties of adipose tissue macrophages recruited during diet-induced obesity. Diabetes. (2007) 56:16–23. 10.2337/db06-1076 17192460

[96] Liu J Divoux A Sun J Zhang J Clement K Glickman JN. Genetic deficiency and pharmacological stabilization of mast cells reduce diet-induced obesity and diabetes in mice. Nat Med. (2009) 15:940–5. 10.1038/nm.1994 19633655 PMC2736875

[97] Winer DA Winer S Shen L Wadia PP Yantha J Paltser G. B cells promote insulin resistance through modulation of T cells and production of pathogenic IgG antibodies. Nat Med. (2011) 17:610–7. 10.1038/nm.2353 21499269 PMC3270885

[98] Schipper HS Rakhshandehroo M van de Graaf SF Venken K Koppen A Stienstra R. Natural killer T cells in adipose tissue prevent insulin resistance. J Clin Invest. (2012) 122:3343–54. 10.1172/JCI62739 22863618 PMC3428087

[99] Nishimura S Manabe I Nagasaki M Eto K Yamashita H Ohsugi M. CD8+ effector T cells contribute to macrophage recruitment and adipose tissue inflammation in obesity. Nat Med. (2009) 15:914–20. 10.1038/nm.1964 19633658

[100] Cipolletta D Kolodin D Benoist C Mathis D. Tissular T(regs): a unique population of adipose-tissue-resident Foxp3+CD4+ T cells that impacts organismal metabolism. Semin Immunol. (2011) 23:431–7. 10.1016/j.smim.2011.06.002 21724410

[101] Bastard JP Jardel C Bruckert E Blondy P Capeau J Laville M. Elevated levels of interleukin 6 are reduced in serum and subcutaneous adipose tissue of obese women after weight loss. J Clin Endocrinol Metab. (2000) 85:3338–42. 10.1210/jcem.85.9.6839 10999830

[102] Ahmed M Gaffen SL. IL-17 in obesity and adipogenesis. Cytokine Growth Factor Rev. (2010) 21:449–53. 10.1016/j.cytogfr.2010.10.005 21084215 PMC3259710

[103] Kimura A Kishimoto T. IL-6: regulator of Treg/Th17 balance. Eur J Immunol. (2010) 40:1830–5. 10.1002/eji.201040391 20583029

[104] Winer S Paltser G Chan Y Tsui H Engleman E Winer D. Obesity predisposes to Th17 bias. Eur J Immunol. (2009) 39:2629–35. 10.1002/eji.200838893 19662632

[105] Lord GM Matarese G Howard JK Baker RJ Bloom SR Lechler RI. Leptin modulates the T-cell immune response and reverses starvation-induced immunosuppression. Nature. (1998) 394:897–901. 10.1038/29795 9732873

[106] Fantuzzi G Faggioni R. Leptin in the regulation of immunity, inflammation, and hematopoiesis. J Leukoc Biol. (2000) 68:437–46. 11037963

[107] La Cava A Alviggi C Matarese G. Unraveling the multiple roles of leptin in inflammation and autoimmunity. J Mol Med. (2004) 82:4–11. 10.1007/s00109-003-0492-1 14556053

[108] Matarese G Di Giacomo A Sanna V Lord GM Howard JK Di Tuoro A. Requirement for leptin in the induction and progression of autoimmune encephalomyelitis. J Immunol. (2001) 166:5909–16. 10.4049/jimmunol.166.10.5909 11342605

[109] Matarese G Sanna V Di Giacomo A Lord GM Howard JK Bloom SR. Leptin potentiates experimental autoimmune encephalomyelitis in SJL female mice and confers susceptibility to males. Eur J Immunol. (2001) 31:1324–32. 10.1002/1521-4141(200105)31:5<1324::AID-IMMU1324>3.0.CO;2-Y 11465089

[110] Reis BS Lee K Fanok MH Mascaraque C Amoury M Cohn LB. Leptin receptor signaling in T cells is required for Th17 differentiation. J Immunol. (2015) 194:5253–60. 10.4049/jimmunol.1402996 25917102 PMC4433844

[111] De Rosa V Procaccini C Cali G Pirozzi G Fontana S Zappacosta S. A key role of leptin in the control of regulatory T cell proliferation. Immunity. (2007) 26:241–55. 10.1016/j.immuni.2007.01.011 17307705

[112] Siegmund B Lehr HA Fantuzzi G. Leptin: a pivotal mediator of intestinal inflammation in mice. Gastroenterology. (2002) 122:2011–25. 10.1053/gast.2002.33631 12055606

[113] Busso N So A Chobaz-Peclat V Morard C Martinez-Soria E Talabot-Ayer D. Leptin signaling deficiency impairs humoral and cellular immune responses and attenuates experimental arthritis. J Immunol. (2002) 168:875–82. 10.4049/jimmunol.168.2.875 11777985

[114] Bernotiene E Palmer G Talabot-Ayer D Szalay-Quinodoz I Aubert ML Gabay C. Delayed resolution of acute inflammation during zymosan-induced arthritis in leptin-deficient mice. Arthritis Res Ther. (2004) 6:R256–63. 10.1186/ar1174 15142272 PMC416449

[115] Matarese G Carrieri PB La Cava A Perna F Sanna V De Rosa V. Leptin increase in multiple sclerosis associates with reduced number of CD4(+)CD25+ regulatory T cells. Proc Natl Acad Sci USA. (2005) 102:5150–5. 10.1073/pnas.0408995102 15788534 PMC555982

[116] Cipolletta D Cohen P Spiegelman BM Benoist C Mathis D. Appearance and disappearance of the mRNA signature characteristic of Treg cells in visceral adipose tissue: age, diet, and PPARgamma effects. Proc Natl Acad Sci USA. (2015) 112:482–7. 10.1073/pnas.1423486112 25550516 PMC4299242

[117] Matarese G La Cava A. The intricate interface between immune system and metabolism. Trends Immunol. (2004) 25:193–200. 10.1016/j.it.2004.02.009 15039046

[118] Procaccini C Galgani M De Rosa V Matarese G. Intracellular metabolic pathways control immune tolerance. Trends Immunol. (2012) 33:1–7. 10.1016/j.it.2011.09.002 22075206

[119] Pearce EL Pearce EJ. Metabolic pathways in immune cell activation and quiescence. Immunity. (2013) 38:633–43. 10.1016/j.immuni.2013.04.005 23601682 PMC3654249

[120] Binger KJ Gebhardt M Heinig M Rintisch C Schroeder A Neuhofer W. High salt reduces the activation of IL-4- and IL-13-stimulated macrophages. J Clin Invest. (2015) 125:4223–38. 10.1172/JCI80919 26485286 PMC4639967

[121] Beier UH Angelin A Akimova T Wang L Liu Y Xiao H. Essential role of mitochondrial energy metabolism in Foxp3(+) T-regulatory cell function and allograft survival. FASEB J. (2015) 29:2315–26. 10.1096/fj.14-268409 25681462 PMC4447222

[122] Gerriets VA Kishton RJ Nichols AG Macintyre AN Inoue M Ilkayeva O. Metabolic programming and PDHK1 control CD4+ T cell subsets and inflammation. J Clin Invest. (2015) 125:194–207. 10.1172/JCI76012 25437876 PMC4382238

[123] Shi LZ Wang R Huang G Vogel P Neale G Green DR. HIF1alpha-dependent glycolytic pathway orchestrates a metabolic checkpoint for the differentiation of TH17 and Treg cells. J Exp Med. (2011) 208:1367–76. 10.1084/jem.20110278 21708926 PMC3135370

[124] He N Fan W Henriquez B Yu RT Atkins AR Liddle C. Metabolic control of regulatory T cell (Treg) survival and function by Lkb1. Proc Natl Acad Sci USA. (2017) 114:12542–7. 10.1073/pnas.1715363114 29109251 PMC5703326

[125] Binger KJ Corte-Real BF Kleinewietfeld M. Immunometabolic regulation of interleukin-17-producing T helper cells: uncoupling new targets for autoimmunity. Front Immunol. (2017) 8:311. 10.3389/fimmu.2017.00311 28377767 PMC5359241

[126] Gerriets VA Kishton RJ Johnson MO Cohen S Siska PJ Nichols AG. Foxp3 and Toll-like receptor signaling balance Treg cell anabolic metabolism for suppression. Nat Immunol. (2016) 17:1459–66. 10.1038/ni.3577 27695003 PMC5215903

[127] Thorens B Mueckler M. Glucose transporters in the 21st Century. Am J Physiol Endocrinol Metab. (2010) 298:E141–5. 10.1152/ajpendo.00712.2009 20009031 PMC2822486

[128] Delgoffe GM Kole TP Zheng Y Zarek PE Matthews KL Xiao B. The mTOR kinase differentially regulates effector and regulatory T cell lineage commitment. Immunity. (2009) 30:832–44. 10.1016/j.immuni.2009.04.014 19538929 PMC2768135

[129] Delgoffe GM Pollizzi KN Waickman AT Heikamp E Meyers DJ Horton MR. The kinase mTOR regulates the differentiation of helper T cells through the selective activation of signaling by mTORC1 and mTORC2. Nat Immunol. (2011) 12:295–303. 10.1038/ni.2005 21358638 PMC3077821

[130] Dang EV Barbi J Yang HY Jinasena D Yu H Zheng Y. Control of T(H)17/T(reg) balance by hypoxia-inducible factor 1. Cell. (2011) 146:772–84. 10.1016/j.cell.2011.07.033 21871655 PMC3387678

[131] Procaccini C De Rosa V Galgani M Abanni L Cali G Porcellini A. An oscillatory switch in mTOR kinase activity sets regulatory T cell responsiveness. Immunity. (2010) 33:929–41. 10.1016/j.immuni.2010.11.024 21145759 PMC3133602

[132] De Rosa V La Cava A Matarese G. Metabolic pressure and the breach of immunological self-tolerance. Nat Immunol. (2017) 18:1190–6. 10.1038/ni.3851 29044230

[133] Huynh A DuPage M Priyadharshini B Sage PT Quiros J Borges CM. Control of PI(3) kinase in Treg cells maintains homeostasis and lineage stability. Nat Immunol. (2015) 16:188–96. 10.1038/ni.3077 25559257 PMC4297515

[134] Shrestha S Yang K Guy C Vogel P Neale G Chi H. Treg cells require the phosphatase PTEN to restrain TH1 and TFH cell responses. Nat Immunol. (2015) 16:178–87. 10.1038/ni.3076 25559258 PMC4297581

[135] Wu D Luo Y Guo W Niu Q Xue T Yang F. Lkb1 maintains Treg cell lineage identity. Nat Commun. (2017) 8:15876. 10.1038/ncomms15876 28621313 PMC5481770

[136] O'Neill HM Lally JS Galic S Thomas M Azizi PD Fullerton MD. AMPK phosphorylation of ACC2 is required for skeletal muscle fatty acid oxidation and insulin sensitivity in mice. Diabetologia. (2014) 57:1693–702. 10.1007/s00125-014-3273-1 24913514

[137] Faubert B Boily G Izreig S Griss T Samborska B Dong Z. AMPK is a negative regulator of the Warburg effect and suppresses tumor growth *in vivo*. Cell Metab. (2013) 17:113–24. 10.1016/j.cmet.2012.12.001 23274086 PMC3545102

[138] Berod L Friedrich C Nandan A Freitag J Hagemann S Harmrolfs K. *De novo* fatty acid synthesis controls the fate between regulatory T and T helper 17 cells. Nat Med. (2014) 20:1327–33. 10.1038/nm.3704 25282359

[139] Raverdeau M Mills KH. Modulation of T cell and innate immune responses by retinoic Acid. J Immunol. (2014) 192:2953–8. 10.4049/jimmunol.1303245 24659788

[140] Lu L Lan Q Li Z Zhou X Gu J Li Q. Critical role of all-trans retinoic acid in stabilizing human natural regulatory T cells under inflammatory conditions. Proc Natl Acad Sci USA. (2014) 111:E3432–40. 10.1073/pnas.1408780111 25099355 PMC4143025

[141] Urry Z Chambers ES Xystrakis E Dimeloe S Richards DF Gabrysova L. The role of 1α,25-dihydroxyvitamin D3 and cytokines in the promotion of distinct Foxp3+ and IL-10+ CD4+ T cells. Eur J Immunol. (2012) 42:2697–708. 10.1002/eji.201242370 22903229 PMC3471131

[142] Chambers ES Suwannasaen D Mann EH Urry Z Richards DF Lertmemongkolchai G. 1α,25-dihydroxyvitamin D3 in combination with transforming growth factor-beta increases the frequency of Foxp3(+) regulatory T cells through preferential expansion and usage of interleukin-2. Immunology. (2014) 143:52–60. 10.1111/imm.12289 24673126 PMC4137955

[143] Nikolouli E Hardtke-Wolenski M Hapke M Beckstette M Geffers R Floess S. Alloantigen-induced regulatory T cells generated in presence of vitamin C display enhanced stability of Foxp3 expression and promote skin allograft acceptance. Front Immunol. (2017) 8:748. 10.3389/fimmu.2017.00748 28702031 PMC5487376

[144] Oyarce K Campos-Mora M Gajardo-Carrasco T Pino-Lagos K. Vitamin C fosters the *in vivo* differentiation of peripheral CD4(+) Foxp3(-) T cells into CD4(+) Foxp3(+) regulatory T cells but impairs their ability to prolong skin allograft survival. Front Immunol. (2018) 9:112. 10.3389/fimmu.2018.00112 29479348 PMC5811461

[145] Baban B Chandler PR Sharma MD Pihkala J Koni PA Munn DH. IDO activates regulatory T cells and blocks their conversion into Th17-like T cells. J Immunol. (2009) 183:2475–83. 10.4049/jimmunol.0900986 19635913 PMC3677163

[146] Yan Y Zhang GX Gran B Fallarino F Yu S Li H. IDO upregulates regulatory T cells via tryptophan catabolite and suppresses encephalitogenic T cell responses in experimental autoimmune encephalomyelitis. J Immunol. (2010) 185:5953–61. 10.4049/jimmunol.1001628 20944000 PMC2998795

[147] Rouse M Singh NP Nagarkatti PS Nagarkatti M. Indoles mitigate the development of experimental autoimmune encephalomyelitis by induction of reciprocal differentiation of regulatory T cells and Th17 cells. Br J Pharmacol. (2013) 169:1305–21. 10.1111/bph.12205 23586923 PMC3831710

[148] He FJ MacGregor GA. A comprehensive review on salt and health and current experience of worldwide salt reduction programmes. J Hum Hypertens. (2009) 23:363–84. 10.1038/jhh.2008.144 19110538

[149] Farquhar WB Edwards DG Jurkovitz CT Weintraub WS. Dietary sodium and health: more than just blood pressure. J Am Coll Cardiol. (2015) 65:1042–50. 10.1016/j.jacc.2014.12.039 25766952 PMC5098396

[150] Farez MF Fiol MP Gaitan MI Quintana FJ Correale J. Sodium intake is associated with increased disease activity in multiple sclerosis. J Neurol Neurosurg Psychiatry. (2015) 86:26–31. 10.1136/jnnp-2014-307928 25168393 PMC12930402

[151] Yang X Yao G Chen W Tang X Feng X Sun L. Exacerbation of lupus nephritis by high sodium chloride related to activation of SGK1 pathway. Int Immunopharmacol. (2015) 29:568–73. 10.1016/j.intimp.2015.09.027 26474695

[152] Wu H Huang X Qiu H Zhao M Liao W Yuan S. High salt promotes autoimmunity by TET2-induced DNA demethylation and driving the differentiation of Tfh cells. Sci Rep. (2016) 6:28065. 10.1038/srep28065 27325182 PMC4914849

[153] Monteleone I Marafini I Dinallo V Di Fusco D Troncone E Zorzi F. Sodium chloride-enriched diet enhanced inflammatory cytokine production and exacerbated experimental colitis in mice. J Crohns Colitis. (2017) 11:237–45. 10.1093/ecco-jcc/jjw139 27473029

[154] Muller DN Wilck N Haase S Kleinewietfeld M Linker RA. Sodium in the microenvironment regulates immune responses and tissue homeostasis. Nat Rev Immunol. (2019) 19:243–54. 10.1038/s41577-018-0113-4 30644452

[155] Wei Y Lu C Chen J Cui G Wang L Yu T. High salt diet stimulates gut Th17 response and exacerbates TNBS-induced colitis in mice. Oncotarget. (2017) 8:70–82. 10.18632/oncotarget.13783 27926535 PMC5352190

[156] Wu C Chen Z Xiao S Thalhamer T Madi A Han T. SGK1 governs the reciprocal development of Th17 and regulatory T cells. Cell Rep. (2018) 22:653–65. 10.1016/j.celrep.2017.12.068 29346764 PMC5826610

[157] Hernandez AL Kitz A Wu C Lowther DE Rodriguez DM Vudattu N. Sodium chloride inhibits the suppressive function of FOXP3+ regulatory T cells. J Clin Invest. (2015) 125:4212–22. 10.1172/JCI81151 26524592 PMC4639983

[158] Sumida T Lincoln MR Ukeje CM Rodriguez DM Akazawa H Noda T. Activated beta-catenin in Foxp3(+) regulatory T cells links inflammatory environments to autoimmunity. Nat Immunol. (2018) 19:1391–402. 10.1038/s41590-018-0236-6 30374130 PMC6240373

[159] Luo Y Xue Y Wang J Dang J Fang Q Huang G. Negligible effect of sodium chloride on the development and function of TGF-beta-induced CD4(+) Foxp3(+) regulatory T cells. Cell Rep. (2019) 26:1869–79 e3. 10.1016/j.celrep.2019.01.066 30759396 PMC6948355

[160] Paling D Solanky BS Riemer F Tozer DJ Wheeler-Kingshott CA Kapoor R. Sodium accumulation is associated with disability and a progressive course in multiple sclerosis. Brain. (2013) 136:2305–17. 10.1093/brain/awt149 23801742

[161] Cortese M Yuan C Chitnis T Ascherio A Munger KL. No association between dietary sodium intake and the risk of multiple sclerosis. Neurology. (2017) 89:1322–9. 10.1212/WNL.0000000000004417 28842447 PMC5649760

[162] Fitzgerald KC Munger KL Hartung HP Freedman MS Montalban X Edan G. Sodium intake and multiple sclerosis activity and progression in BENEFIT. Ann Neurol. (2017) 82:20–9. 10.1002/ana.24965 28556498 PMC5555227

[163] Haase S Wilck N Kleinewietfeld M Muller DN Linker RA. Sodium chloride triggers Th17 mediated autoimmunity. J Neuroimmunol. (2019) 329:9–13. 10.1016/j.jneuroim.2018.06.016 29983198

[164] Lloyd-Price J Abu-Ali G Huttenhower C. The healthy human microbiome. Genome Med. (2016) 8:51. 10.1186/s13073-016-0307-y 27122046 PMC4848870

[165] Macpherson AJ Harris NL. Interactions between commensal intestinal bacteria and the immune system. Nat Rev Immunol. (2004) 4:478–85. 10.1038/nri1373 15173836

[166] Kamada N Seo SU Chen GY Nunez G. Role of the gut microbiota in immunity and inflammatory disease. Nat Rev Immunol. (2013) 13:321–35. 10.1038/nri3430 23618829

[167] Belkaid Y Hand TW. Role of the microbiota in immunity and inflammation. Cell. (2014) 157:121–41. 10.1016/j.cell.2014.03.011 24679531 PMC4056765

[168] Lathrop SK Bloom SM Rao SM Nutsch K Lio CW Santacruz N. Peripheral education of the immune system by colonic commensal microbiota. Nature. (2011) 478:250–4. 10.1038/nature10434 21937990 PMC3192908

[169] Atarashi K Tanoue T Oshima K Suda W Nagano Y Nishikawa H. Treg induction by a rationally selected mixture of Clostridia strains from the human microbiota. Nature. (2013) 500:232–6. 10.1038/nature12331 23842501

[170] Geuking MB Cahenzli J Lawson MA Ng DC Slack E Hapfelmeier S. Intestinal bacterial colonization induces mutualistic regulatory T cell responses. Immunity. (2011) 34:794–806. 10.1016/j.immuni.2011.03.021 21596591

[171] Arpaia N Campbell C Fan X Dikiy S van der Veeken J deRoos P. Metabolites produced by commensal bacteria promote peripheral regulatory T-cell generation. Nature. (2013) 504:451–5. 10.1038/nature12726 24226773 PMC3869884

[172] Kim KS Hong SW Han D Yi J Jung J Yang BG. Dietary antigens limit mucosal immunity by inducing regulatory T cells in the small intestine. Science. (2016) 351:858–63. 10.1126/science.aac5560 26822607

[173] Haghikia A Jorg S Duscha A Berg J Manzel A Waschbisch A. Dietary fatty acids directly impact central nervous system autoimmunity via the small intestine. Immunity. (2015) 43:817–29. 10.1016/j.immuni.2015.09.007 26488817

[174] Poutahidis T Kleinewietfeld M Smillie C Levkovich T Perrotta A Bhela S. Microbial reprogramming inhibits Western diet-associated obesity. PLoS ONE. (2013) 8:e68596. 10.1371/journal.pone.0068596 23874682 PMC3707834

[175] Smith PM Howitt MR Panikov N Michaud M Gallini CA Bohlooly YM. The microbial metabolites, short-chain fatty acids, regulate colonic Treg cell homeostasis. Science. (2013) 341:569–73. 10.1126/science.1241165 23828891 PMC3807819

[176] Furusawa Y Obata Y Fukuda S Endo TA Nakato G Takahashi D. Commensal microbe-derived butyrate induces the differentiation of colonic regulatory T cells. Nature. (2013) 504:446–50. 10.1038/nature12721 24226770

[177] Atarashi K Tanoue T Shima T Imaoka A Kuwahara T Momose Y. Induction of colonic regulatory T cells by indigenous Clostridium species. Science. (2011) 331:337–41. 10.1126/science.1198469 21205640 PMC3969237

[178] Wu HJ Ivanov II Darce J Hattori K Shima T Umesaki Y. Gut-residing segmented filamentous bacteria drive autoimmune arthritis via T helper 17 cells. Immunity. (2010) 32:815–27. 10.1016/j.immuni.2010.06.001 20620945 PMC2904693

[179] Ivanov II Atarashi K Manel N Brodie EL Shima T Karaoz U. Induction of intestinal Th17 cells by segmented filamentous bacteria. Cell. (2009) 139:485–98. 10.1016/j.cell.2009.09.033 19836068 PMC2796826

[180] Gaboriau-Routhiau V Rakotobe S Lecuyer E Mulder I Lan A Bridonneau C. The key role of segmented filamentous bacteria in the coordinated maturation of gut helper T cell responses. Immunity. (2009) 31:677–89. 10.1016/j.immuni.2009.08.020 19833089

[181] Luu M Pautz S Kohl V Singh R Romero R Lucas S. The short-chain fatty acid pentanoate suppresses autoimmunity by modulating the metabolic-epigenetic crosstalk in lymphocytes. Nat Commun. (2019) 10:760. 10.1038/s41467-019-08711-2 30770822 PMC6377655

[182] Häger J Bang H Hagen M Frech M Trager P Sokolova MV. The role of dietary fiber in rheumatoid arthritis patients: a feasibility study. Nutrients. (2019) 11:e2392. 10.3390/nu11102392 31591345 PMC6836071

[183] Smits HH Engering A van der Kleij D de Jong EC Schipper K van Capel TM Selective probiotic bacteria induce IL-10-producing regulatory T cells *in vitro* by modulating dendritic cell function through dendritic cell-specific intercellular adhesion molecule 3-grabbing non-integrin. J Allergy Clin Immunol. (2005) 115:1260–7. 10.1016/j.jaci.2005.03.036 15940144

[184] Kwon HK Lee CG So JS Chae CS Hwang JS Sahoo A. Generation of regulatory dendritic cells and CD4+Foxp3+ T cells by probiotics administration suppresses immune disorders. Proc Natl Acad Sci USA. (2010) 107:2159–64. 10.1073/pnas.0904055107 20080669 PMC2836639

[185] David LA Maurice CF Carmody RN Gootenberg DB Button JE Wolfe BE. Diet rapidly and reproducibly alters the human gut microbiome. Nature. (2014) 505:559–63. 10.1038/nature12820 24336217 PMC3957428

[186] De Filippo C Cavalieri D Di Paola M Ramazzotti M Poullet JB Massart S. Impact of diet in shaping gut microbiota revealed by a comparative study in children from Europe and rural Africa. Proc Natl Acad Sci USA. (2010) 107:14691–6. 10.1073/pnas.1005963107 20679230 PMC2930426

[187] Wu GD Chen J Hoffmann C Bittinger K Chen YY Keilbaugh SA. Linking long-term dietary patterns with gut microbial enterotypes. Science. (2011) 334:105–8. 10.1126/science.1208344 21885731 PMC3368382

[188] De Luca F Shoenfeld Y. The microbiome in autoimmune diseases. Clin Exp Immunol. (2019) 195:74–85. 10.1111/cei.13158 29920643 PMC6300652

[189] Nogueira AR Shoenfeld Y. Microbiome and autoimmune diseases: cause and effect relationship. Curr Opin Rheumatol. (2019) 31:471–4. 10.1097/BOR.0000000000000628 31192811

[190] Cekanaviciute E Yoo BB Runia TF Debelius JW Singh S Nelson CA. Gut bacteria from multiple sclerosis patients modulate human T cells and exacerbate symptoms in mouse models. Proc Natl Acad Sci USA. (2017) 114:10713–8. 10.1073/pnas.1711235114 28893978 PMC5635915

[191] Willebrand R Hamad I Van Zeebroeck L Kiss M Bruderek K Geuzens A. High salt inhibits tumor growth by enhancing anti-tumor immunity. Front Immunol. (2019) 10:1141. 10.3389/fimmu.2019.01141 31214164 PMC6557976

[192] Cignarella F Cantoni C Ghezzi L Salter A Dorsett Y Chen L. Intermittent fasting confers protection in CNS autoimmunity by altering the gut microbiota. Cell Metab. (2018) 27:1222–35 e6. 10.1016/j.cmet.2018.05.006 29874567 PMC6460288

[193] Morton AM Sefik E Upadhyay R Weissleder R Benoist C Mathis D. Endoscopic photoconversion reveals unexpectedly broad leukocyte trafficking to and from the gut. Proc Natl Acad Sci USA. (2014) 111:6696–701. 10.1073/pnas.1405634111 24753589 PMC4020091

[194] Esplugues E Huber S Gagliani N Hauser AE Town T Wan YY. Control of TH17 cells occurs in the small intestine. Nature. (2011) 475:514–8. 10.1038/nature10228 21765430 PMC3148838

[195] Long SA Buckner JH. CD4+FOXP3+ T regulatory cells in human autoimmunity: more than a numbers game. J Immunol. (2011) 187:2061–6. 10.4049/jimmunol.1003224 21856944 PMC3160735

